# Controlled Orientation of Active Sites in a Nanostructured Multienzyme Complex

**DOI:** 10.1038/srep39587

**Published:** 2016-12-22

**Authors:** Sung In Lim, Byungseop Yang, Younghan Jung, Jaehyun Cha, Jinhwan Cho, Eun-Sil Choi, Yong Hwan Kim, Inchan Kwon

**Affiliations:** 1 Department of Chemical Engineering, University of Virginia, VA 22904, United States; 2 School of Materials Science and Engineering, Gwangju Institute of Science and Technology (GIST), Gwangju, 61005, Republic of Korea; 3 Department of Biological Sciences, College of Natural Sciences, Chonnam National University, Gwangju, 61186, Republic of Korea; 4 School of Energy and Chemical Engineering, Ulsan National Institute of Science and Technology (UNIST), Ulsan, 44919, Republic of Korea

## Abstract

Multistep cascade reactions in nature maximize reaction efficiency by co-assembling related enzymes. Such organization facilitates the processing of intermediates by downstream enzymes. Previously, the studies on multienzyme nanocomplexes assembled on DNA scaffolds demonstrated that closer interenzyme distance enhances the overall reaction efficiency. However, it remains unknown how the active site orientation controlled at nanoscale can have an effect on multienzyme reaction. Here, we show that controlled alignment of active sites promotes the multienzyme reaction efficiency. By genetic incorporation of a non-natural amino acid and two compatible bioorthogonal chemistries, we conjugated mannitol dehydrogenase to formate dehydrogenase with the defined active site arrangement with the residue-level accuracy. The study revealed that the multienzyme complex with the active sites directed towards each other exhibits four-fold higher relative efficiency enhancement in the cascade reaction and produces 60% more D-mannitol than the other complex with active sites directed away from each other.

Multiple enzymes involved in related cellular processes are co-localized or clustered to form large non-covalent assemblies, thereby increasing local concentrations of intermediates near active sites^1^,^2^,^3^,^4^. Engineering efforts to build enzymatic cascade reactions with enhanced catalytic efficiency have proven enzyme clustering as a successful approach. A variety of spatially organized multienzyme structures have been developed using nucleic acids and proteins as a scaffold^5^,^6^,^7^,^8^. The computer-aided model quantitatively demonstrated the benefits of rapid processing of intermediates by co-localized enzymes^9^.

Nano-scale scaffolding technologies have enabled the creation of complex, organized multienzyme structures with spatial programmability. As a pioneering work, Fu *et al*. conjugated enzymes to a single-stranded DNA, and arranged them through hybridization onto DNA origami tiles with defined interenzyme position and spacing^10^,^11^. By systematically varying the distance between enzymes, they showed the multienzyme reaction displayed the distance-dependent efficiency, and attained great improvements when the enzymes were closely spaced as little as 10 nm.

Another important question on multienzyme systems is whether active site orientation affects cascade reaction efficiency. To date, the importance of enzyme orientation in multienzyme systems has been proposed by the computational simulation^12^,^13^, but not yet demonstrated experimentally due to the technical hurdle of controlling the spatial arrangement of catalytic sites between paired enzymes in solution^14^. In case of the multienzyme systems constructed using nucleic acids or protein scaffolds^7^,^11^, the active site of each enzyme had random or unidirectional orientations, hindering investigation of catalytic benefits from active sites directed towards one another in multienzyme cascade reactions. Furthermore, immobilization of multiple enzyme pairs on such scaffolds limits accurate measurement of orientation effect on overall reaction efficiency enhancement because of multiple targets in proximity accessible for intermediates, the attraction between scaffolds and intermediates, and increased stability of intermediates^15^,^16^. Although combination of residue-specific incorporation of a non-natural amino acid and bioorthogonal click chemistry was used to construct multi-enzyme complexes^17^, there was still some restriction in choosing coupling sites to control active site orientation. Genetic fusion of related enzymes was used to demonstrate the proximity-induced substrate channeling, but not applicable to studies of orientation dependence due to limited control of geometry and alignment^18^,^19^. In this study, we described catalytic benefits from the controlled orientation of active sites of enzymes interacting in a cascade reaction. To this end, we used site-specific incorporation of a reactive non-natural amino acid (NNAA) into enzymes followed by bioorthogonal enzyme-to-enzyme conjugation. During the ribosomal synthesis, NNAAs containing a bioorthogonal group can be incorporated into a target protein via expanded genetic codes and engineered translational machinery^20^,^21^,^22^. One of great advantages is that a single or multiple NNAA(s) can be introduced to any defined position in the primary sequence of a protein, thereby permitting site-specific and chemoselective bioconjugation with other functional entities^23^,^24^,^25^. We employed two bioorthogonal reactions in this study. In strain-promoted azide-alkyne cycloaddition (SPAAC), an internal alkyne activated by ring-strained energy drives cycloaddition with an azide at room temperature without any catalyst. SPAAC has high degree of bioorthogonality without harmful byproducts, which is suitable for protein conjugation involving chemically sensitive enzymes and metal-binding proteins^26^,^27^,^28^,^29^. Inverse electron demand Diels-Alder reaction (IEDDA) was recently introduced into the chemistry pool for site-specific protein conjugation^30^,^31^. IEDDA is the cycloaddition between tetrazine and strained alkene, and has the fastest reaction rate in a range of 10^4^–10^5^ M^−1^s^−1^, thus proceeding efficiently even at low concentrations. In addition, IEDDA features orthogonality towards SPAAC as well as natural functionalities in proteins, enabling one-pot dual labeling and construction of spatially controlled multienzyme conjugate^31^,^32^.

Through combined use of SPAAC and IEDDA, we generated two chemically cross-linked multienzyme conjugates that were identical except for the active site orientation. We hypothesized that the active sites directed towards each other (face-to-face orientation) facilitate the intermediate transfer between enzymes more efficiently than the active sites directed away from each other (back-to-back orientation) (Fig. 1a).

## Results and Discussion

### Design and genetic incorporation of AZF into FDH and MDH

**Figure 1:**
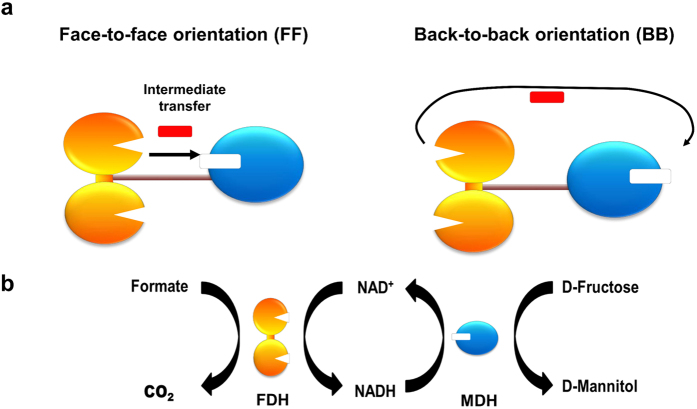
Schematic diagrams of orientation variants of the multienzyme nanocomplex and the coupled catalytic reaction. (**a**) The active sites directed towards each other (face-to-face orientation) and active sites directed away from each other (back-to-back orientation) in the multienzyme complex consisting of dimeric formate dehydrogenase (FDH; orange color) and monomeric mannitol dehydrogenase (MDH; blue color). (**b**) The enzymatic cascade reactions converting fructose to mannitol by MDH using cofactors supplied from formate conversion into CO_2_ by FDH.

Formate dehydrogenase (FDH) used in this study originates from *Thiobacillus* and is a homodimer with a molecular mass of 45 kDa for a single subunit^33^,^34^. Its dimeric form in solution was confirmed by size exclusion chromatography (Supplementary Fig. 1). FDH is a NAD-dependent oxidoreductase that has 10^3^ times higher activity of formate oxidation to carbon dioxide than the reverse reaction^33^, and useful for enzymatic regeneration of nicotinamide cofactors^35^. Mannitol dehydrogenase (MDH) used in this study originates from *Pseudomonas fluorescens* and is a monomer with a molecular mass of 55 kDa, as revealed in size exclusion chromatography (Supplementary Fig. 1). MDH is a NAD-dependent oxidoreductase that has 2.5 times higher activity of D-fructose reduction to D-mannitol than the reverse reaction^36^, and can be used for biocatalytic production of D-mannitol through coupling with a cofactor regeneration system employing FDH or glucose dehydrogenase^37^. In the cascade reaction consisting of FDH and MDH (Fig. 1b), NADH is regenerated by FDH-mediated formate oxidation, thereby continuously fueling MDH-mediated D-mannitol production. D-fructose reduction to D-mannitol by MDH is kinetically more efficient than formate conversion to CO_2_ by FDH^33^,^38^. Therefore, in the presence of excess substrates for both enzymes (formate and D-fructose), the transfer rate of NADH from FDH to MDH is supposed to govern the overall efficiency of the cascade reaction. Pazmino *et al*. nicely showed that fusion of two enzymes can be used to efficiently recycle cofactors^39^, though the active site orientation was not controlled. We also previously demonstrated that the induced internal diffusion of NADH between the active sites enhanced specific pathway flux and product titers^31^.

**Figure 2:**
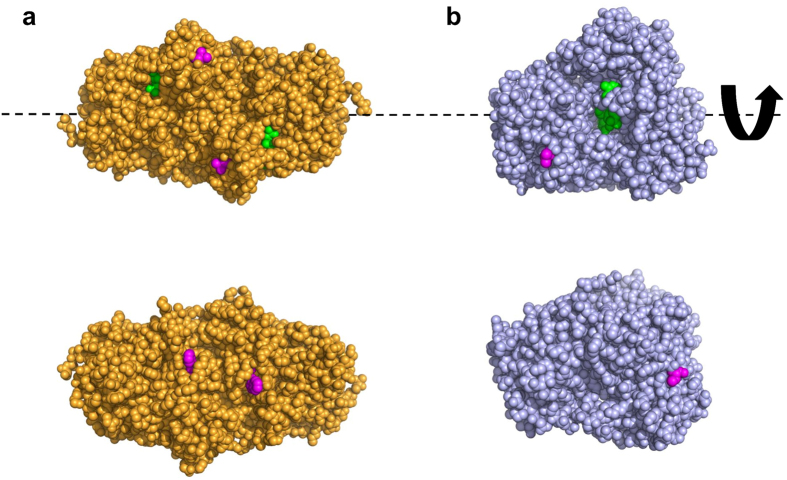
Graphical representation of three-dimensional structures of FDH and MDH. (**a**) A dimeric form of FDH in complex with a cofactor (green) was derived from Protein Data Bank (PDB ID: 3WR5). AZF incorporation sites, V237 (top) and W172 (bottom), are highlighted in magenta. (**b**) MDH in complex with a cofactor (green) was derived from Protein Data Bank (PDB ID: 1LJ8). AZF incorporation sites, V417 (top) and V271 (bottom), are highlighted in magenta. Images at the bottom of (**a**,**b)** were obtained by rotating images at the top by 180 degrees about the horizontal axis.

To investigate how the relative orientation of active sites affects the substrate transfer efficiency, we designed FDH-MDH conjugates with defined orientation of each enzyme. Depending on the site of NNAA incorporation and the structure of a chemical linker, spatial orientation of a single protein towards a specific target as well as the geometry of macroscopic protein assembly can be controlled and customized in accordance with user-defined preferences^40^,^41^,^42^. As a first step to construct FDH-MDH conjugates with their active sites in face-to-face (FF conjugate) or back-to-back orientation (BB conjugate) (Fig. 1a), we chose two surface-exposed positions in FDH and MDH, respectively, for site-specific genetic incorporation of *p*-azidophenylalanine (AZF). For FDH, valine at position 237 (V237) is in close proximity to the NAD^+^-binding site on the same side (Fig. 2a, top), while tryptophan at position 172 (W172) is on the opposite side, away from the binding site (Fig. 2a, bottom). For MDH, V417 and V271 are counterparts to V237 and W172 of FDH, respectively. V417 is near the NADH-binding site (Fig. 2b, top), while V271 is positioned on the opposite side of the NADH-binding site (Fig. 2b, bottom).

To genetically encode AZF, we introduced amber codons into predetermined sites in the coding sequence of FDH and MDH. *E. coli* equipped with an orthogonal pair of amber suppressor tRNA and aminoacyl-tRNA synthetase produced AZF-bearing proteins in culture medium containing AZF. Following the purification of FDH and MDH variants, we performed both fluorescent dye labeling and mass spectrometry to verify the bioorthogonal reactivity and site-specificity of AZF. While the wild-type FDH and MDH exhibited no fluorescence when mixed with a DBCO-functionalized dye, variants emitted strong fluorescence (Supplementary Fig. 2a). MALDI-TOF mass spectra of tryptic fragments demonstrated high-fidelity incorporation of AZF in response to the amber codons at position 172 and 237 for FDH and position 271 and 417 for MDH, respectively (Supplementary Fig. 2b, Supplementary Table 1, and Supplementary Table 2). We assessed the effect of AZF incorporation on the catalytic activity in comparison to the wild type (Supplementary Fig. 3). Whereas MDH variants retained the enzymatic activity comparable to that of the wild type, AZF incorporation into FDH slightly affected the native activity, leading to less than 6% reduction or 3% increase in *k*_cat_/*K*_m_ (Supplementary Table 3). Similarly, MDH variants containing AZF exhibited about 4 or 5% lower *k*_cat_/*K*_m_ values compared to that of wild-type MDH (Supplementary Table 3).

### Synthesis and characterization of FDH-MDH conjugates

**Figure 3:**
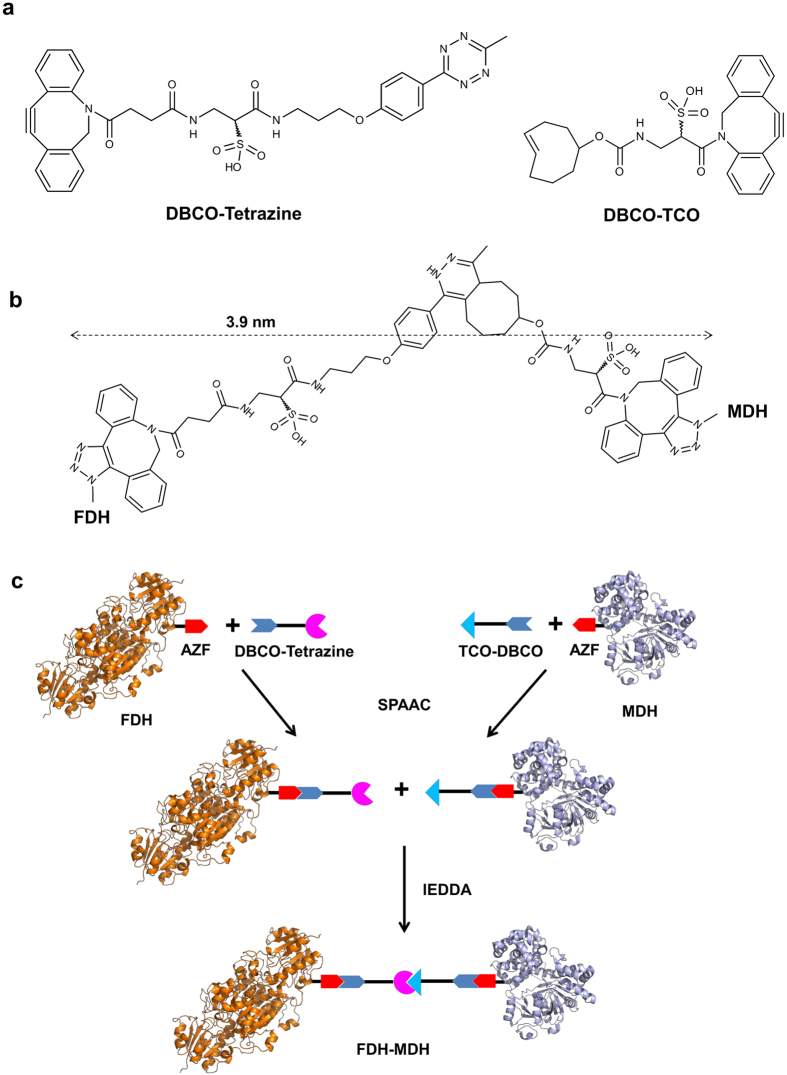
Construction of the FDH-MDH complex. (**a**) DBCO-derivatized bifunctional linkers. (**b**) The entire structure and length of the conjugated chemical linker bridging FDH and MDH. (**c**) Schematic diagram showing construction of the FDH-MDH complex via consecutive click reactions. The DBCO group reacts with AZF incorporated into FDH and MDH via SPAAC. The conjugation between FDH and MDH is mediated by IEDDA between tetrazine and TCO.

Site-specific incorporation of AZF and its chemoselective reactivity provided a modular platform to generate FDH-MDH conjugates with controlled spatial orientation of their active sites. Crosslinking of FDH-V237AZF with MDH-V417AZF could have their cofactor binding sites permanently facing towards each other, namely, the FF conjugate (Fig. 1a, left). Likewise, conjugation of FDH-W172AZF with MDH-V271AZF could generate the BB conjugate in which active sites were directed away from each other (Fig. 1a, right). To synthesize the FF conjugate, we conjugated FDH-V237AZF to the DBCO-Tetrazine linker (Fig. 3a) through SPAAC between AZF and DBCO, and removed residual linkers by desalting to give FDH-V237TET. Similarly, we attached the DBCO-TCO linker to MDH-V417AZF (Fig. 3a) to obtain MDH-V417TCO. We assessed the effect of a linker conjugation on the catalytic activity in comparison to unconjugated variants in the concentration range (0 to 300 μM) of cofactors used in characterization of FDH-MDH conjugates (Supplementary Fig. 3). Conjugation of a tetrazine linker resulted in about 15% increase in *k*_cat_/*K*_m_ value of FDH-V237 variant, but led to about 20% reduction in *k*_cat_/*K*_m_ value of FDH-W172 compared to each corresponding FDH variant without a linker (Supplementary Table 3). However, when the concentration of substrate NAD^+^ is above 150 μM, both FDH-W172TET and FDH-V237TET exhibited comparable reaction rates (Supplementary Fig. 3a). Therefore, at 200 μM of NAD^+^ used in the cascade reactions, we believed that the linker conjugation to FDH variants did not substantially contribute to the difference in the multienzyme reaction efficiency depending on the active site orientation.

Next, IEDDA between tetrazine and TCO covalently cross-linked FDH-V237TET to MDH-V417TCO to yield the FF conjugate. In SDS-PAGE analysis of the reaction mixture, we identified a new single band slightly larger than the 110 kDa-standard protein, indicating the TCO group specifically reacted with the tetrazine functionality (Supplementary Fig. 4). The overall yields of the conjugation process were comparable to those reported previously^31^. We performed the two-step liquid chromatography to isolate the FF conjugate. First, the anion exchanger removed unreacted FDH-V237TET from the mixture (Supplementary Fig. 5a). Then, the size exclusion chromatography successfully separated the FF conjugate from unreacted MDH-V417TCO (Supplementary Fig. 5b). We applied the same reaction scheme and the purification method to obtain the BB conjugate by the crosslink between FDH-W172AZF and MDH-V271AZF. We could define the interenzyme distance between FDH and MDH based upon the length of the entire chemical bridge (3.9 nm, Fig. 3b).

A molar composition of FDH-MDH conjugates could be either one or two MDH molecule(s) per FDH dimer, since FDH has two AZFs due to its dimeric form. The elution profile of conjugates observed in the size exclusion chromatogram was a single peak in symmetry, indicating the conjugate mostly consisted of a single species (Supplementary Fig. 5b). To quantitatively evaluate the molar composition under denaturing condition, both FF and BB conjugates were subjected to a standard SDS-PAGE which resolved the unconjugated FDH monomer (Supplementary Fig. 6, lower band) and the FDH monomer conjugated to MDH via the chemical linker (Supplementary Fig. 6, upper band). Densitometric analyses of each conjugate revealed the same molar composition, two molar equivalents of FDH monomer with respect to MDH. These data demonstrated that a dimeric FDH cross-linked to a single MDH was a major species in a native state for both conjugates.

### Effect of active site orientation on the multienzyme reaction efficiency

We investigated the importance of active site orientation on the overall reaction efficiency in the multienzyme cascade. In the presence of a saturating amount of formate, D-fructose, and NAD^+^, but without NADH, efficient transfer of NADH from the binding site of FDH to that of MDH is a rate-limiting step to produce D-mannitol (Fig. 1b). We designed a spectrophotometric method in which the NADH transfer efficiency (ε) defined as the rate of NADH oxidation by MDH over the rate of NADH production by FDH was measured by performing two independent assays and then combining them as detailed in Supplementary Information. In NADH-mediated enzymatic reactions, the spectrophotometric reading for NADH oxidation (ΔOD_340nm_) is a standard way calculating catalytic efficiency. In the FDH-MDH multienzyme cascade, however, it’s not feasible to accurately measure the NADH oxidation rate by reading ΔOD_340nm_ due to continuous generation of NADH by FDH. A higher ε value indicates more efficient transfer of NADH from FDH to MDH. The FF conjugate exhibited higher ε values in all conjugate concentrations than the BB conjugate (Supplementary Fig. 8). Furthermore, in comparison to the BB conjugate, the FF conjugate facilitated more efficient NADH transfer at lower conjugate concentrations. These results indicate that active sites aligned in the face-to-face orientation channeled the NADH flux between them while reducing the release of NADH into solution.

**Figure 4:**
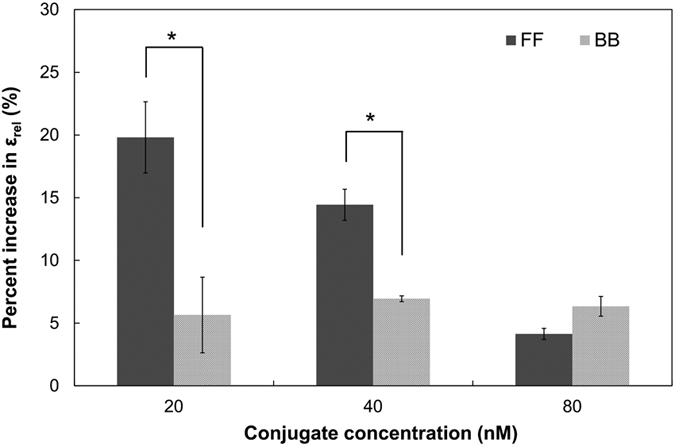
Increase in the relative efficiency of the NADH transfer (ε_rel_) for the FF and BB conjugates. The stoichiometry of dimeric FDH and MDH in free enzyme reactions was 1.1:1 which corresponded to the molar composition of FDH-MDH conjugates. Mean ± s.e.m. n = 3. *p < 0.05 (Two-tailed Student’s t test).

In order to demonstrate catalytic benefits of constructing the nanostructured multienzyme complex with the face-to-face orientation, we measured the relative efficiency of the NADH transfer, ε_rel_, defined as the ratio of ε for the FF or BB conjugate over ε for unconjugated FDH and MDH mix. A ε_rel_ value greater than 1.0 indicates enhanced reaction efficiency of the FDH-MDH conjugate relative to the free enzymes. We found that both conjugates had enhanced reaction efficiency in comparison to the free enzymes (Fig. 4). Remarkably, the FF conjugate showed four- and two-fold greater increase in the reaction efficiency than the BB conjugate at 20 and 40 nM, respectively, while there was no significant difference in the reaction efficiency between the FF and BB conjugates at 80 nM (Fig. 4). We speculate that this is because the FF conjugate retained NADHs in a confined region between the active site of each enzyme and had a higher chance of NADH oxidation by MDH. On the other hand, NADH in the BB conjugate had a lower probability of oxidation because NADH had to diffuse first into solution and then back to the active site of the partnered MDH or other MDH nearby. This notion is supported by Brownian dynamics simulations that orientation of active sites favorable for intermediate transfer, in particular the face-to-face arrangement, facilitated intermediate transfer to the downstream enzyme by limiting free diffusion of intermediates into the bulk solution^12^. The ε_rel_ values for the BB conjugate remained consistent at 1.05 (5% increase). The constant ε_rel_ values for the BB conjugate in the concentration of 20 nM to 80 nM suggest that its improved reaction efficiency originated from the closer interenzyme distance fixed by the chemical linker in comparison to distantly spaced free enzymes.

Next, we investigated whether the FF conjugate produces more D-mannitol than the BB conjugate using the D-mannitol production protocols reported previously^31^. The FF and BB conjugates produced 36.3 ± 7.7 and 21.9 ± 1.6 μM of D-mannitol, respectively. The FF conjugate produced significantly more D-mannitol (about 60%) than the BB conjugate (p < 0.05, two-tailed Student’s t test).

## Conclusion

Technical challenges in engineering an orientation-controlled multienzyme complex have been overcome by employing the position-specific incorporation of NNAAs and chemoselective bioconjugation chemistries. By covalently coupling two enzymes, FDH and MDH, with geometric control, we could quantitatively measure enhancement in activity of the cascade reaction depending on the active site orientation. The overall reaction efficiency enhancement was more effective when the active sites were directed towards each other than the active sites directed away from each other. For the first time, our results provided the experimental evidence of the orientation-dependent acceleration of the multienzyme reaction, and may aid the design of spatially organized multienzyme nanostructures with improved performance.

## Methods

### Materials

DBCO-PEG_4_-carboxyrhodamine 110, DBCO-amine, TCO-NHS ester, and tetrazine-DBCO were purchased from Bioconjugate Technology Company (Scottsdale, AZ). *p*-Azido-L-phenylalanine (AZF) was purchased from Chem-Impex International (Wood Dale, IL) and dissolved in 0.2 M sodium hydroxide to prepare 100 mM stock solution. Ni-NTA agarose and pQE80 plasmid were obtained from Qiagen (Valencia, CA). Vivaspin centrifugal concentrators with a MWCO of 50 kDa were obtained from Sartorius Corporation (Bohemia, NY). ZipTip C18 was purchased from Millipore Corporation (Billerica, MA). Sequencing-grade modified trypsin was obtained from Promega Corporation (Madison, WI). PD-10 desalting columns and Superdex 200 10/300 GL sized exclusion column were obtained from GE Healthcare (Piscataway, NJ). UNO Q1 anion exchange column and Biologic DuoFlow chromatography system were obtained from Bio-Rad (Hercules, CA). All chemicals were obtained from Sigma-Aldrich Corporation (St. Louis, MO) unless otherwise stated.

### Plasmids and bacterial strains

A plasmid pEVOL-pAzF^43^ encoding a mutant pair of tyrosyl-tRNA synthetase/amber suppressor tRNA derived from *Methanococcus jannaschii* was a generous gift from Dr. Peter Schultz in Scripps Institute (Addgene plasmid # 31186). The *fdh* gene encoding formate dehydrogenase originating from *Thiobacillus* sp. KNK65MA was PCR amplified with a C-terminal hexahistidine sequence using pET-23b(+)-TsFDH as a template^33^, and subcloned into pQE80 plasmid to obtain pQE80-FDH. Site-directed mutagenic PCR was performed to introduce an amber codon (UAG) in place of a tryptophan codon at positions 172 or valine codon at position 237, yielding pQE80-FDH-W172amb and pQE80-FDH-V237amb, respectively. *E. coli* TOP10 was transformed with pQE80-FDH for the expression of the wild-type FDH (FDH-WT), affording TOP10 [FDH]. To express AZF-incorporated FDH (FDH-W172AZF or FDH-V237AZF), *E. coli* C321.ΔA.exp^44^ genomically optimized for amber suppression was a gift from George Church (Addgene plasmid # 49018), and co-transformed with pEVOL-pAzF and pQE80-FDH-W172amb or pQE80-FDH-V237amb, affording C321.ΔA.exp [FDH-W172amb] and C321.ΔA.exp [FDH-V237amb], respectively. The *mdh* gene encoding mannitol-2-dehydrogenase derived from *Pseudomonas fluorescens*^36^ was synthesized by GenScript (Piscataway, NJ), and subcloned with an additional C-terminal hexahistidine sequence into pQE80 to generate pQE80-MDH. Site-directed mutagenic PCR was performed to introduce an amber codon in place of a valine codon at positions 271 or 417, yielding pQE80-MDH-V271amb and pQE80-MDH-V417amb, respectively. *E. coli* TOP10 was transformed with pQE80-MDH for expression of the wild-type MDH (MDH-WT), yielding TOP10 [MDH]. To express AZF-incorporated MDH (MDH-V271AZF or MDH-V417AZF), *E. coli* C321.ΔA.exp was co-transformed with pEVOL-pAzF and pQE80-MDH-V271amb or pQE80-MDH-V417amb, yielding C321.ΔA.exp [MDH-V271amb] and C321.ΔA.exp [MDH-V417amb], respectively. All DNA cloning in this study used the restriction-free cloning method^45^.

### Site-specific incorporation of AZF into FDH or MDH

The overnight culture of C321.ΔA.exp [FDH-W172amb, FDH-V237amb, MDH-V271amb, or MDH-V417amb] was inoculated into fresh 2 × YT medium containing 100 μg/mL ampicillin and 35 μg/mL chloramphenicol at 1:100 (v/v) dilution, and was grown with shaking (220 rpm) at 37 °C. When the OD_600_ reached 0.5, AZF was added to a final concentration of 1 mM. Following the temperature shift to 30 °C after 10 min, the protein expression was induced by 1 mM IPTG and 0.2% (w/v) *L*-(+)-arabinose. After 12 h, cells were harvested and pelleted by centrifugation at 4,000 g for 10 min before storage at −80 °C. To extract and purify FDH or MDH containing AZF, cell pellets were resuspended with the lysis buffer consisting of 50 mM sodium phosphate (pH 7.5), 0.3 M NaCl, 10 mM imidazole, 1 mg/mL lysozyme, 10 μg/mL DNase, 5 μg/mL RNase, and protease inhibitor cocktail, and mixed by rotation at 37 °C for 1 h. After centrifugation at 10,000 g for 30 min, the clear supernatant was recovered, mixed with Ni-NTA agarose for 1 h at 4 °C, and then washed with the washing buffer consisting of 50 mM sodium phosphate (pH 7.5), 0.3 M NaCl, and 20 mM imidazole on a gravity-flow column. Proteins were eluted by the elution buffer consisting of 50 mM sodium phosphate (pH 7.5), 0.3 M NaCl, and 250 mM imidazole, and then buffer-exchanged to an appropriate buffer by a PD-10 desalting column. FDH-WT or MDH-WT was similarly expressed and purified, except that TOP10 [FDH or MDH] was used as an expression host without adding AZF and *L*-(+)-arabinose.

### MALDI-TOF mass spectrometry

Trypsin was added to protein at 0.5 mg/mL in PBS to a final ratio trypsin : protein ratio of 1:20 (w/w). After incubation at 37 °C overnight, the digestion was quenched by adding 0.1% (v/v) trichloroacetic acid, and then desalted on a ZipTip C18 according to the manufacturer’s protocol. Purified tryptic digests mixed with DHB matrix (20 mg/mL of 2,5-dihydroxybenzoic acid and 2 mg/mL of *L*-(−)-fucose dissolved in 10% ethanol) at 1:1 (v/v) were analyzed by Microflex MALDI-TOF M/S (Bruker Corporation, Billerica, MA).

### Site-specific dye labeling by SPAAC

Four molar excess of DBCO- PEG_4_-carboxyrhodamine 110 (120 μM) was reacted with proteins (30 μM) in PBS at RT for 2 h, and then analyzed by SDS-PAGE to measure in-gel fluorescence in BioSpectrum imaging system (UVP, Upland, CA). The gel was irradiated by λ_ex_ = 480 nm, and light emitted above 510 nm was captured.

### Enzyme kinetics

Enzymatic reduction of NAD^+^ to NADH by FDH-WT and its variants was measured by monitoring increase in A_340nm_. The reaction was initiated by mixing FDH (100 nM) or variants with sodium formate (50 mM) and various concentrations of NAD^+^ in PBS. Enzymatic oxidation of NADH to NAD^+^ by MDH-WT and its variants was measured by monitoring decrease in A_340nm_. The reaction was initiated by mixing MDH (50 nM) or variants with fructose (50 mM) and various concentrations of NADH in PBS. All measurements were made in triplicate at 25 °C in a standard 96-well plate on the Synergy^TM^ four multimode microplate reader (BioTek, Winooski, VT). Reaction rates were determined by dividing the slope (min^−1^, OD change in the first one minute upon initiation) by the molar extinction coefficient of NADH, 6,220 M^−1^cm^−1^, and the path length of 0.52 cm. The Michaelis-Menten equation was applied to obtain kinetic constants.

### Synthesis of FDH-MDH Conjugates

FDH-W172AZF or FDH-V237AZF was mixed with 4 molar excess of DBCO-Tetrazine in PBS containing 5% (v/v) DMSO and reacted at RT for 7 h. Residual DBCO-Tetrazine was removed by desalting and buffered-exchanging to 20 mM bis-tris buffered at pH 6.0 on a PD-10 column. MDH-V271AZF or MDH-V417AZF was similarly treated except that DBCO-TCO was used instead of DBCO-Tetrazine. DBCO-TCO was prepared by mixing 2.5 molar excess of TCO-NHS with DBCO-amine in DMSO at RT for 1.5 h followed by excess tris (pH 9.0) to quench. FDH-W172TET and MDH-V271TCO were mixed at 1:1 molar stoichiometry, and reacted at a total protein concentration of 5 mg/mL at RT for 2 h. The reaction mixture was loaded onto an anion exchange column, UNO Q1, pre-equilibrated with 20 mM bis-tris (pH 6.0), and resolved by applying a NaCl gradient. A fraction containing the conjugate and unreacted MDH-V271TCO was collected and resolved on a size exclusion column, Superdex 200, to isolate pure FDH-MDH. The conjugation of FDH-V237TET to MDH-V417TCO was performed in the same way.

### Characterization of FDH-MDH conjugates

Molar compositions of FDH-MDH conjugates were analyzed by band intensities seen in SDS-PAGE using Image J–a public domain Java image-processing program. Based on 1:1 molar ratio of FDH monomer conjugated to MDH and unconjugated FDH monomer, the molar absorption coefficient of FDH-MDH conjugates was calculated using ExPASy ProtParam tool (http://web.expasy.org/protparam) with the input amino acid sequence obtained by combining MDH sequence (MDH monomer) and FDH sequence twice (FDH dimer), and found to be 180,030 M^−1^cm^−1^, which was used to determine concentrations of FDH-MDH conjugates throughout the enzymatic assays in this study.

### Measurement of cascade enzymatic reactions

Details are described in the Supplementary Information.

## Additional Information

**How to cite this article**: Lim, S. I. *et al*. Controlled Orientation of Active Sites in a Nanostructured Multienzyme Complex. *Sci. Rep.* **6**, 39587; doi: 10.1038/srep39587 (2016).

**Publisher's note:** Springer Nature remains neutral with regard to jurisdictional claims in published maps and institutional affiliations.

## Supplementary Material

Supplementary Information

## References

[b1] An S., Kumar R., Sheets E. D. & Benkovic S. J. Reversible compartmentalization of de novo purine biosynthetic complexes in living cells. Science 320, 103–106 (2008). 18388293 10.1126/science.1152241

[b2] Leibundgut M., Maier T., Jenni S. & Ban N. The multienzyme architecture of eukaryotic fatty acid synthases. Curr Opin Struct Biol 18, 714–725 (2008). 18948193 10.1016/j.sbi.2008.09.008

[b3] Narayanaswamy R. . Widespread reorganization of metabolic enzymes into reversible assemblies upon nutrient starvation. Proc Natl Acad Sci USA 106, 10147–10152 (2009). 19502427 10.1073/pnas.0812771106 PMC2691686

[b4] Schmeing T. M. & Ramakrishnan V. What recent ribosome structures have revealed about the mechanism of translation. Nature 461, 1234–1242 (2009). 19838167 10.1038/nature08403

[b5] Conrado R. J. . DNA-guided assembly of biosynthetic pathways promotes improved catalytic efficiency. Nucleic Acids Res 40, 1879–1889 (2012). 22021385 10.1093/nar/gkr888 PMC3287197

[b6] Delebecque C. J., Lindner A. B., Silver P. A. & Aldaye F. A. Organization of intracellular reactions with rationally designed RNA assemblies. Science 333, 470–474 (2011). 21700839 10.1126/science.1206938

[b7] Dueber J. E. . Synthetic protein scaffolds provide modular control over metabolic flux. Nat Biotechnol 27, 753–759 (2009). 19648908 10.1038/nbt.1557

[b8] Moon T. S., Dueber J. E., Shiue E. & Prather K. L. Use of modular, synthetic scaffolds for improved production of glucaric acid in engineered E. coli. Metab Eng 12, 298–305 (2010). 20117231 10.1016/j.ymben.2010.01.003

[b9] Castellana M. . Enzyme clustering accelerates processing of intermediates through metabolic channeling. Nat Biotechnol 32, 1011–1018 (2014). 25262299 10.1038/nbt.3018 PMC4666537

[b10] Fu J., Liu M., Liu Y., Woodbury N. W. & Yan H. Interenzyme substrate diffusion for an enzyme cascade organized on spatially addressable DNA nanostructures. J Am Chem Soc 134, 5516–5519 (2012). 22414276 10.1021/ja300897h PMC3319985

[b11] Fu J. . Multi-enzyme complexes on DNA scaffolds capable of substrate channelling with an artificial swinging arm. Nat Nanotechnol 9, 531–536 (2014). 24859813 10.1038/nnano.2014.100

[b12] Bauler P., Huber G., Leyh T. & McCammon J. A. Channeling by Proximity: The Catalytic Advantages of Active Site Colocalization Using Brownian Dynamics. J Phys Chem Lett 1, 1332–1335 (2010). 20454551 10.1021/jz1002007 PMC2865391

[b13] Roberts C. C. & Chang C.-e. A. Modeling of Enhanced Catalysis in Multienzyme Nanostructures: Effect of Molecular Scaffolds, Spatial Organization, and Concentration. Journal of Chemical Theory and Computation 11, 286–292 (2015). 26574226 10.1021/ct5007482

[b14] Lin J.-L., Palomec L. & Wheeldon I. Design and Analysis of Enhanced Catalysis in Scaffolded Multienzyme Cascade Reactions. ACS Catalysis 4, 505–511 (2014).

[b15] Zhang Y., Ge J. & Liu Z. Enhanced Activity of Immobilized or Chemically Modified Enzymes. ACS Catalysis 5, 4503–4513 (2015).

[b16] Idan O. & Hess H. Origins of activity enhancement in enzyme cascades on scaffolds. ACS Nano 7, 8658–8665 (2013). 24007359 10.1021/nn402823k

[b17] Schoffelen S., Beekwilder J., Debets M. F., Bosch D. & van Hest J. C. Construction of a multifunctional enzyme complex via the strain-promoted azide-alkyne cycloaddition. Bioconjug Chem 24, 987–996 (2013). 23713411 10.1021/bc400021j

[b18] Pettersson H. & Pettersson G. Kinetics of the coupled reaction catalysed by a fusion protein of beta-galactosidase and galactose dehydrogenase. Biochim Biophys Acta 1549, 155–160 (2001). 11690652 10.1016/s0167-4838(01)00252-7

[b19] Seo H. S. . Characterization of a bifunctional enzyme fusion of trehalose-6-phosphate synthetase and trehalose-6-phosphate phosphatase of Escherichia coli. Appl Environ Microbiol 66, 2484–2490 (2000). 10831428 10.1128/aem.66.6.2484-2490.2000 PMC110565

[b20] Hoesl M. G. & Budisa N. *In vivo* incorporation of multiple noncanonical amino acids into proteins. Angew Chem Int Ed Engl 50, 2896–2902 (2011). 21404373 10.1002/anie.201005680

[b21] Johnson J. A., Lu Y. Y., Van Deventer J. A. & Tirrell D. A. Residue-specific incorporation of non-canonical amino acids into proteins: recent developments and applications. Curr Opin Chem Biol 14, 774–780 (2010). 21071259 10.1016/j.cbpa.2010.09.013 PMC3008400

[b22] Brustad E. M. & Arnold F. H. Optimizing non-natural protein function with directed evolution. Curr Opin Chem Biol 15, 201–210 (2011). 21185770 10.1016/j.cbpa.2010.11.020 PMC3080047

[b23] Kim C. H., Axup J. Y. & Schultz P. G. Protein conjugation with genetically encoded unnatural amino acids. Curr Opin Chem Biol 17, 412–419 (2013). 23664497 10.1016/j.cbpa.2013.04.017 PMC4284959

[b24] Lim S. I. & Kwon I. Bioconjugation of therapeutic proteins and enzymes using the expanded set of genetically encoded amino acids. Crit Rev Biotechnol, 1–13 (2015). 10.3109/07388551.2015.1048504 26036278

[b25] Seo M. H. . Controlled and oriented immobilization of protein by site-specific incorporation of unnatural amino acid. Anal Chem 83, 2841–2845 (2011). 21428414 10.1021/ac103334b

[b26] Agard N. J., Prescher J. A. & Bertozzi C. R. A strain-promoted [3 + 2] azide-alkyne cycloaddition for covalent modification of biomolecules in living systems. J Am Chem Soc 126, 15046–15047 (2004). 15547999 10.1021/ja044996f

[b27] Lim S. I., Hahn Y. S. & Kwon I. Site-specific albumination of a therapeutic protein with multi-subunit to prolong activity *in vivo*. J Control Release 207, 93–100 (2015). 25862515 10.1016/j.jconrel.2015.04.004 PMC4430413

[b28] Kim Y., Kim S. H., Ferracane D., Katzenellenbogen J. A. & Schroeder C. M. Specific labeling of zinc finger proteins using noncanonical amino acids and copper-free click chemistry. Bioconjug Chem 23, 1891–1901 (2012). 22871171 10.1021/bc300262h PMC3462365

[b29] Lim S. I., Yoon S., Kim Y. H. & Kwon I. Site-specific bioconjugation of an organometallic electron mediator to an enzyme with retained photocatalytic cofactor regenerating capacity and enzymatic activity. Molecules 20, 5975–5986 (2015). 25853315 10.3390/molecules20045975 PMC6272604

[b30] Blackman M. L., Royzen M. & Fox J. M. Tetrazine ligation: fast bioconjugation based on inverse-electron-demand Diels-Alder reactivity. J Am Chem Soc 130, 13518–13519 (2008). 18798613 10.1021/ja8053805 PMC2653060

[b31] Lim S. I., Cho J. & Kwon I. Double clicking for site-specific coupling of multiple enzymes. Chem Commun (Camb) 51, 13607–13610 (2015). 26191550 10.1039/c5cc04611d

[b32] Sachdeva A., Wang K., Elliott T. & Chin J. W. Concerted, rapid, quantitative, and site-specific dual labeling of proteins. J Am Chem Soc 136, 7785–7788 (2014). 24857040 10.1021/ja4129789 PMC4333588

[b33] Choe H. . Efficient CO2-reducing activity of NAD-dependent formate dehydrogenase from Thiobacillus sp. KNK65MA for formate production from CO2 gas. PLoS One 9, e103111 (2014). 25061666 10.1371/journal.pone.0103111 PMC4111417

[b34] Nanba H., Takaoka Y. & Hasegawa J. Purification and characterization of an alpha-haloketone-resistant formate dehydrogenase from Thiobacillus sp. strain KNK65MA, and cloning of the gene. Biosci Biotechnol Biochem 67, 2145–2153 (2003). 14586102 10.1271/bbb.67.2145

[b35] Weckbecker A., Groger H. & Hummel W. Regeneration of nicotinamide coenzymes: principles and applications for the synthesis of chiral compounds. Adv Biochem Eng Biotechnol 120, 195–242 (2010). 20182929 10.1007/10_2009_55

[b36] Brunker P., Altenbuchner J., Kulbe K. D. & Mattes R. Cloning, nucleotide sequence and expression of a mannitol dehydrogenase gene from Pseudomonas fluorescens DSM 50106 in Escherichia coli. Biochim Biophys Acta 1351, 157–167 (1997). 9116029 10.1016/s0167-4781(96)00189-3

[b37] Slatner M., Nagl G., Haltrich D., Kulbe K. D. & Nidetzky B. Enzymatic production of pure D-mannitol at high productivity. Biocatal Biotransfor 16, 351–363 (1998).

[b38] Bubner P., Klimacek M. & Nidetzky B. Structure-guided engineering of the coenzyme specificity of Pseudomonas fluorescens mannitol 2-dehydrogenase to enable efficient utilization of NAD(H) and NADP(H). FEBS Lett 582, 233–237 (2008). 18082142 10.1016/j.febslet.2007.12.008

[b39] Torres Pazmino D. E. . Self-sufficient Baeyer-Villiger monooxygenases: effective coenzyme regeneration for biooxygenation by fusion engineering. Angew Chem Int Ed Engl 47, 2275–2278 (2008). 18224639 10.1002/anie.200704630

[b40] Guan D., Kurra Y., Liu W. & Chen Z. A click chemistry approach to site-specific immobilization of a small laccase enables efficient direct electron transfer in a biocathode. Chem Commun (Camb) 51, 2522–2525 (2015). 25566975 10.1039/c4cc09179e

[b41] Hutchins B. M. . Site-specific coupling and sterically controlled formation of multimeric antibody fab fragments with unnatural amino acids. J Mol Biol 406, 595–603 (2011). 21237172 10.1016/j.jmb.2011.01.011 PMC4278757

[b42] Lim S. I., Mizuta Y., Takasu A., Kim Y. H. & Kwon I. Site-specific bioconjugation of a murine dihydrofolate reductase enzyme by copper(I)-catalyzed azide-alkyne cycloaddition with retained activity. PLoS One 9, e98403 (2014). 24887377 10.1371/journal.pone.0098403 PMC4041766

[b43] Chin J. W. . Addition of p-azido-L-phenylalanine to the genetic code of Escherichia coli. J Am Chem Soc 124, 9026–9027 (2002). 12148987 10.1021/ja027007w

[b44] Lajoie M. J. . Genomically recoded organisms expand biological functions. Science 342, 357–360 (2013). 24136966 10.1126/science.1241459 PMC4924538

[b45] Bond S. R. & Naus C. C. RF-Cloning.org: an online tool for the design of restriction-free cloning projects. Nucleic Acids Res 40, W209–213 (2012). 22570410 10.1093/nar/gks396 PMC3394257

