# Supplementary material for: Controlled Orientation of Active Sites in a Nanostructured Multienzyme Complex
Source: Sci Rep. 2016 Dec 22;6:39587. doi: 10.1038/srep39587 (PMC5177890; doi:10.1038/srep39587)
Supplement: Supplementary Information [file srep39587-s1.pdf]

**Controlled Orientation of Active Sites in a Nanostructured Multienzyme Complex**

Sung In Lim[1,#], Byungseop Yang[2,#], Younghan Jung[2], Jaehyun Cha[2], Jinhwan Cho[2], Eun-Sil Choi[2,3], Yong Hwan Kim[4], & Inchan Kwon[1,2,*]

[1]Department of Chemical Engineering, University of Virginia, VA 22904, United States; [2]School of Materials Science and Engineering, Gwangju Institute of Science and Technology (GIST), Gwangju 61005, Republic of Korea; [3]Department of Biological Sciences, College of Natural Sciences, Chonnam National University, Gwangju 61186, Republic of Korea; [4]School of Energy and Chemical Engineering, Ulsan National Institute of Science and Technology (UNIST), Ulsan 44919, Republic of Korea

[#]These authors contributed equally to this work.

*Corresponding author

Address: School of Materials Science and Engineering, Gwangju Institute of Science and Technology, Gwangju 61005, Republic of Korea

Tel.: +82 62-715-2312; Fax: +82 62-715-2304

E-mail:inchan@gist.ac.kr (I. Kwon)

# Supplementary Information

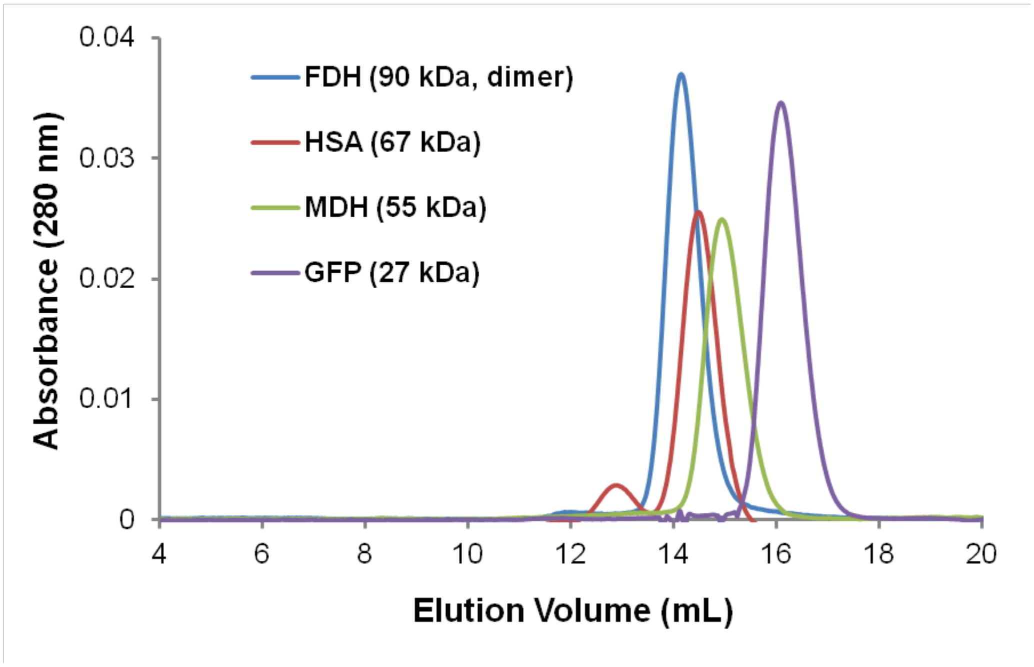


**Figure 1. Elution profiles of FDH, MDH, and standard proteins in size exclusion chromatography.**

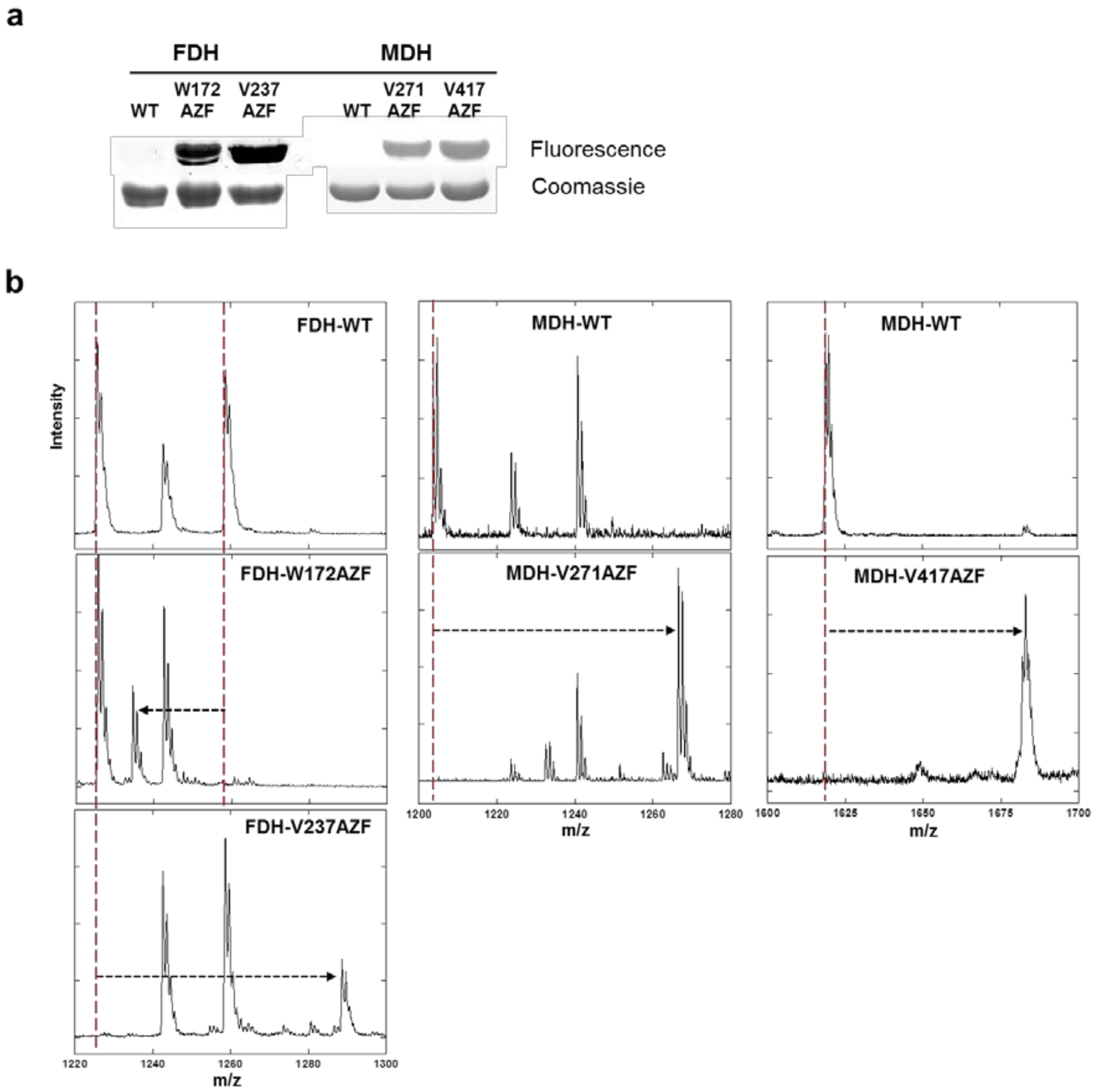


**Figure 2. Validation of bioorthogonal reactivity and site-specificity of genetically incorporated AZF. a**. In-gel fluorescence analyses of FDH, MDH, and their variants upon completion of reactions with DBCO-PEG$_4$-carboxyrhodamine. The gel was subjected to UV (390 nm) irradiation to excite the fluorophore (fluorescence panel), and then stained with Coomassie blue (Coomassie panel) to visualize proteins. **b**, MALDI-TOF MS analyses of trypsin-digested FDH, MDH, and their variants.

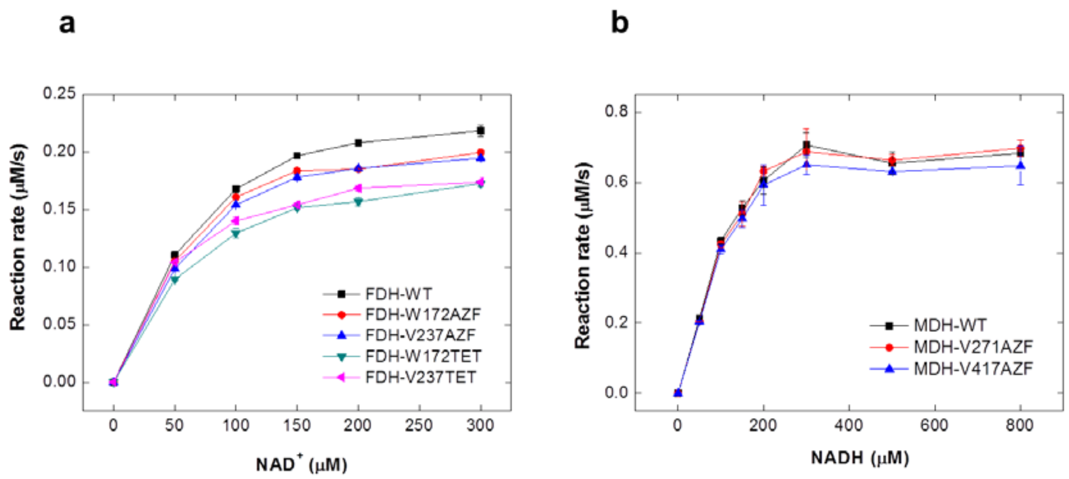


**Figure 3. Enzymatic activity of FDH, MDH, and their variants. a**, Rates of formate oxidation to carbon dioxide by FDH and variants were plotted as a function of various concentration of a cofactor. **b**, Rates of D-fructose reduction to D-mannitol by MDH and variants were plotted as a function of various concentration of a cofactor. Error bars represent standard deviations of the experimental results performed in triplicate. Error bars, which are smaller than size of symbols, are not shown.

**Table 1. MALDI-TOF MS analyses of trypsin-digested FDH variants**

| | NYIPSHD-$W_{172}$-AR | | NYIPSHD-$AZF_{172}$-AR | | ELGL-$V_{237}$-WHDTR | | ELGL-$AZF_{237}$-WHDTR | |
|---|---|---|---|---|---|---|---|---|
| | Expected | Observed | Expected[a] | Observed | Expected | Observed | Expected[a] | Observed |
| FDH_WT | 1258.6 | 1258.67 | - | - | 1225.63 | 1225.75 | - | - |
| FDH_W172AZF | - | - | 1234.6 | 1234.6 | | | | |
| FDH_V237AZF | - | - | | | | | 1288.68 | 1288.57 |

[a]Conversion of azide to amine upon laser irradiation

**Table 2. MALDI-TOF MS analyses of trypsin-digested MDH variants**

| | F-$W_{271}$-NGRPAWEK | | F-$AZF_{271}$_NGRPAWEK | | G-$V_{417}$-DENGVSYTIPDPR | | G-$AZF_{417}$-DENGVSYTIPDPR | |
|---|---|---|---|---|---|---|---|---|
| | Expected | Observed | Expected[a] | Observed | Expected | Observed | Expected[a] | Observed |
| MDH_WT | 1203.63 | 1203.64 | - | - | 1618.78 | 1618.71 | - | - |
| MDH_W271AZF | - | - | 1266.67 | 1266.58 | | | | |
| MDH_V417AZF | - | - | | | | | 1681.82 | 1681.71 |

[a]Conversion of azide to amine upon laser irradiation

**Table 3. Kinetic parameters of FDH and its variants**

| | $k_{cat}$ ($s^{-1}$) | $K_m$ (μM) | $k_{cat}$ / $K_m$ ($s^{-1}$ $μM^{-1}$) |
|---|---|---|---|
| FDH-WT | 2.8 ± 0.1 | 67 ± 6 | 0.043 |
| FDH-W172AZF | 2.4 ± 0.1 | 58 ± 6 | 0.041 |
| FDH-V237AZF | 2.4 ± 0.1 | 64 ± 6 | 0.038 |
| FDH-W172TET | 2.1 ± 0.1 | 65 ± 5 | 0.032 |
| FDH-V237TET | 2.0 ± 0.03 | 47 ± 3 | 0.043 |
| MDH-WT | 81 ± 6.0 | 88 ± 23 | 0.92 |
| MDH-V417AZF | 83 ± 6.0 | 95 ± 24 | 0.87 |
| MDH-V271AZF | 77 ± 5.4 | 88 ± 22 | 0.88 |

Values ± s.e.m of regression obtained by fitting to the Michaelis-Menten equation

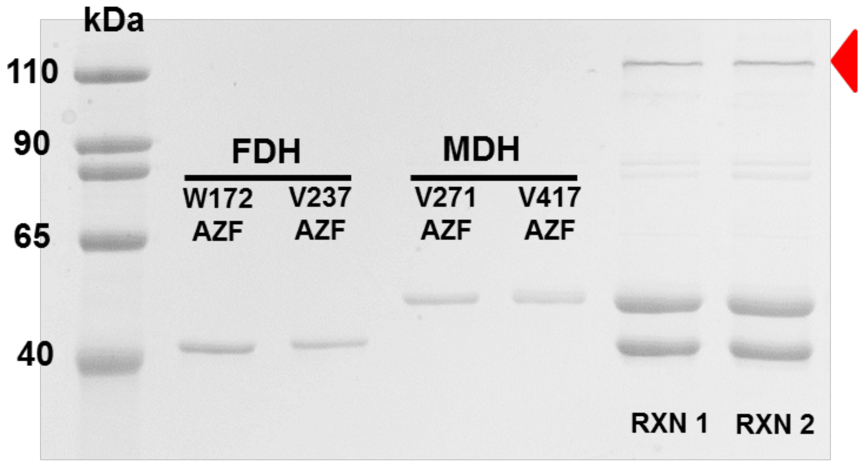


RXN1: FDH-V237Tetrazine + MDH-V417TCO
RNX2: FDH-W172Tetazine + MDH-V271TCO

**Figure 4. SDS-PAGE analysis of FDH and MDH variants and conjugation reaction mixtures. The bands of FDH-MDH conjugates are marked by a red triangle.**

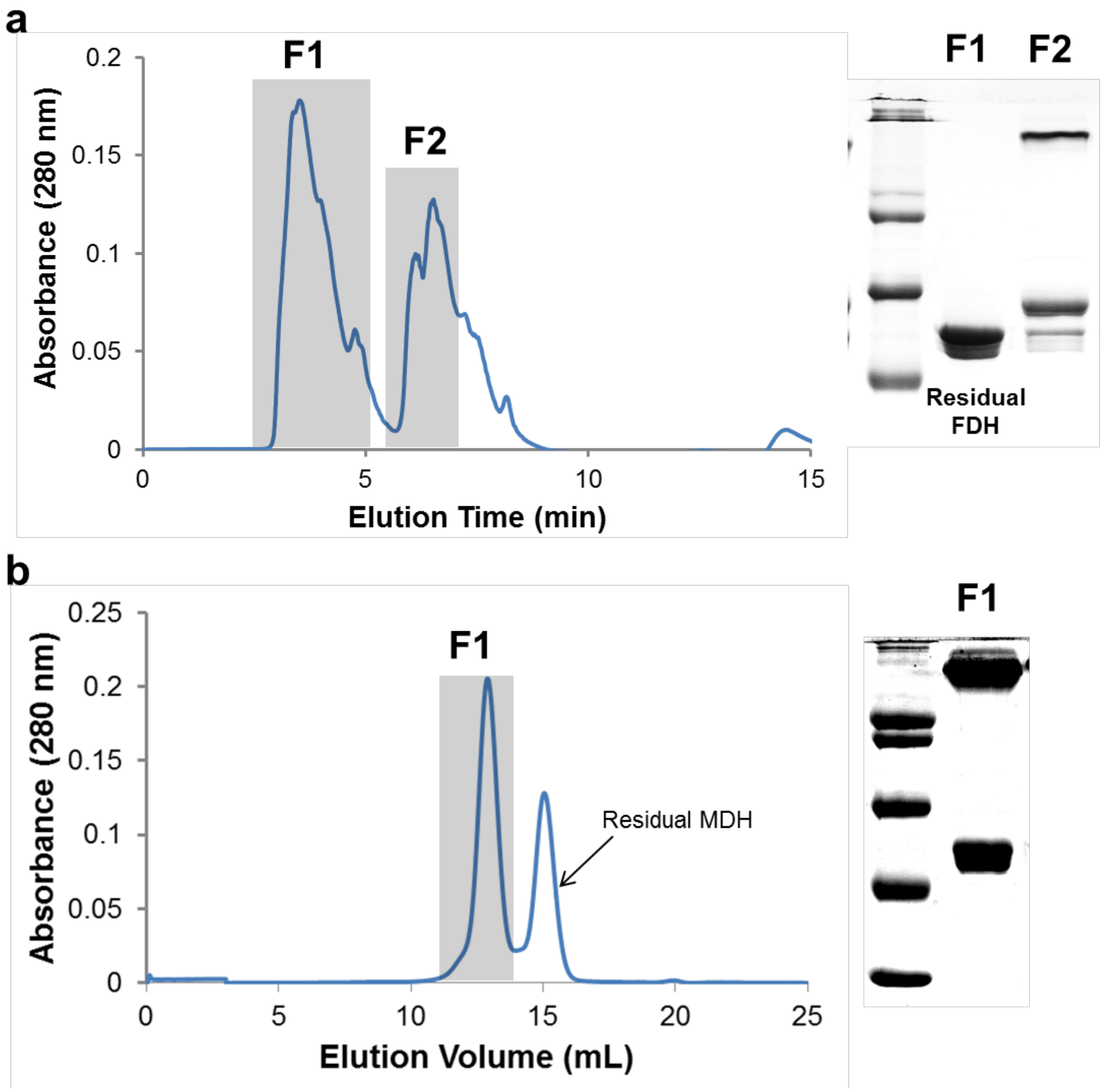


**Figure 5. Purification of FDH-MDH conjugates by liquid chromatography. a**, Anion exchange chromatography to eliminate residual FDHs (F1) and SDS-PAGE of eluting fractions. **b**, Size exclusion chromatography of F2 obtained from anion exchange chromatography to isolate the pure FDH-MDH conjugate (F1) from residual MDHs.

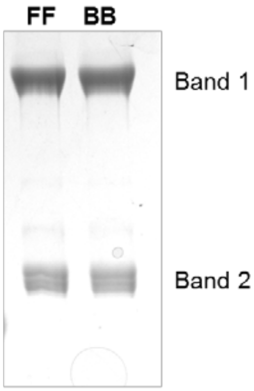


| Conjugate | Band | Identity | Intensity (a.u.) | MW (Da) | Mole (Intensity / MW, a.u.) | Mole (rel.) |
|---|---|---|---|---|---|---|
| FDH237-MDH417 (FF) | 1 | FDH monomer conjugated to MDH | 16,995 | 100,160 | 0.17 | 1.0 |
| | 2 | FDH monomer | 8,557 | 44,780 | 0.19 | 1.1 |
| FDH172-MDH271 (BB) | 1 | FDH monomer conjugated to MDH | 15,740 | 100,160 | 0.16 | 1.0 |
| | 2 | FDH monomer | 8,223 | 44,780 | 0.18 | 1.1 |

**Figure 6. Densitometric determination of molar stoichiometry of FDH-MDH conjugates.** The gel image was analyzed by Image J to estimate band intensity. The gel image was analyzed by Image J to estimate band intensity. The MW of the monomeric FDH-MDH conjugate (100,160 Da) is the sum of MWs of monomeric FDH and MDH. Molar composition of each species was calculated assuming that the band intensity is proportional to the amount of protein,

**Determination of NADH processing rate and efficiency in enzyme cascade reactions**

The control assay that measures an initial rate of NADH production by FDH activity in the absence of NADH consumption by MDH activity was initiated by mixing 10 µL of FDH-MDH conjugate at an appropriate molar concentration with 190 µL of the assay buffer (50 mM sodium formate, 200 µM $NAD^+$ in PBS) which lacks D-fructose—a substrate of MDH. The cascade assay that measures an initial rate of NADH production in the presence of NADH consumption by MDH activity was initiated by mixing 10 µL of FDH-MDH conjugate at an appropriate molar concentration with 190 µL of the assay buffer (50 mM sodium formate, 50 mM D-fructose, 200 µM $NAD^+$ in PBS) which includes D-fructose. Increase in absorbance at 340 nm of the control assay or the cascade assay was monitored upon initiation to obtain NADH production rates defined as the OD change in the first one minute: $\Delta A_{cont}$ [$min^{-1}$] for the control assay and $\Delta A_{casc}$ [$min^{-1}$] for the cascade assay (Figure 7). In the control assay, the enzymatic reaction was performed in a solution devoid of D-fructose to measure the NADH production rate solely by FDH ($\Delta A_{cont}$). In the cascade assay, the same reaction was performed in the presence of all components required for the coupled reaction to measure the NADH production rate affected by the NADH consumption by MDH ($\Delta A_{casc}$). If the processing of NADH by MDH became faster due to its increased local concentration contributed by favorable orientation of the active site, it would lead to decrease in $\Delta A_{casc}$ (Equation 1).

$$\Delta A_{cont}\ [min^{-1}] = \text{Rate of NADH production by FDH}$$

$$\Delta A_{casc}\ [min^{-1}] = \text{Rate of NADH production by FDH} - \text{Rate of NADH consumption by MDH} \quad (1)$$

The efficiency of the NADH processing, ε, is defined as

$$\varepsilon = \frac{\text{Rate of NADH consumption by MDH}}{\text{Rate of NADH production by FDH}} = \frac{\text{Rate of NADH production by FDH} - \Delta\text{Acasc}}{\text{Rate of NADH production by FDH}} = 1 - \frac{\Delta\text{Acasc}}{\Delta\text{Acont}} \qquad (2)$$

All measurements were made in triplicate at 25˚C in a standard 96-well plate on the Synergy$^{TM}$ four multimode microplate reader. Similarly, both the control and the cascade assay were performed with free enzymes instead of FDH-MDH conjugates; 1.1 molar equivalent of dimeric FDH-V237AZF mixed with monomeric MDH-V417AZF or FDH-172AZF with MDH-V271AZF corresponding to the molar composition of FDH-MDH conjugates as revealed in Fig. 6. Next, to investigate if the NADH channeling relative to random Brownian diffusion of NADH provides enhanced reaction efficiency, ε of free enzymes ($\varepsilon_{free}$) was compared with that of the conjugate ($\varepsilon_{FF}$ or $\varepsilon_{BB}$) to yield the relative efficiency defined as follows: $\varepsilon_{rel} = \varepsilon_{FF} / \varepsilon_{free}$ or $\varepsilon_{BB} / \varepsilon_{free}$ where FF stands for FDH-V237AZF conjugated to MDH-V417AZF and BB for FDH-W172AZF conjugated to MDH-271AZF.

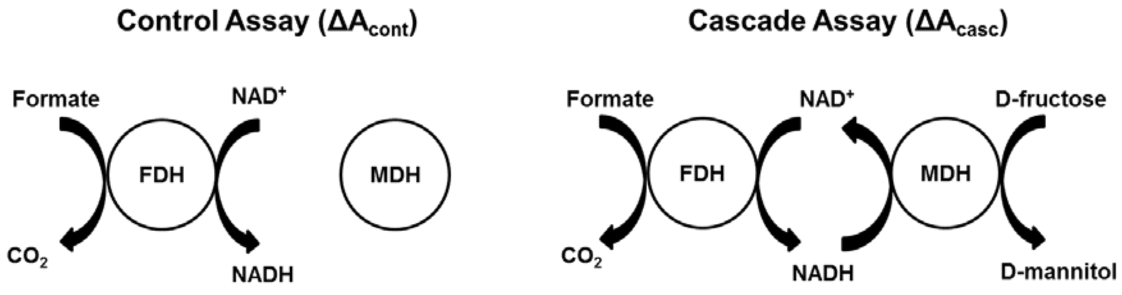


**Figure 7. Measurement of the NADH production in the absence or presence of MDH activity.**

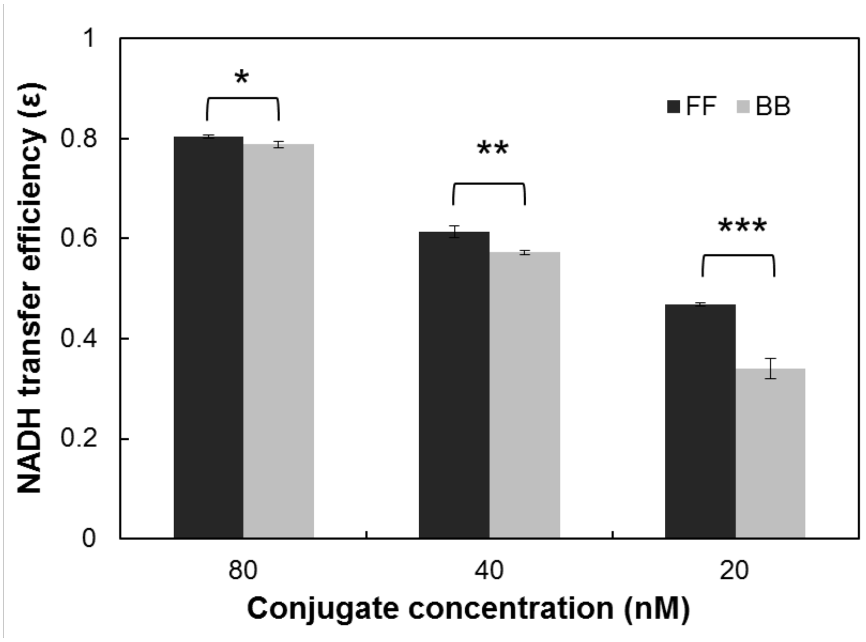


**Figure 8. NADH transfer efficiency (ε) at various concentrations of conjugates.** The absorbance change at 340 nm was measured for 1 min after initiation of the control assay and the cascade assay, separately, and used to calculate ε using the equation (2). Mean ± s.e.m. n = 3. p values (Two-tailed Student's t test): *, 0.133; **, 0.0394; ***, 0.0242.
